# Aha! Ratings as Metacognitive Judgments

**DOI:** 10.3390/jintelligence14090198

**Published:** 2026-09-01

**Authors:** Jennifer Wiley, Taylor Strickland Miller

**Affiliations:** Department of Psychology, University of Illinois at Chicago, Chicago, IL 60607, USA

**Keywords:** Aha!, insight, restructuring, problem solving, monitoring judgments, growth mindset

## Abstract

There has been an increase in studies using Aha! ratings to explore insight in creative problem solving. Aha! moments are often assumed to serve as a marker for insight—meaning that solutions are achieved via restructuring. However, similar to metacognitive paradigms that collect FOKs (Feelings of Knowing) or JOLs (Judgments of Learning) and JOUs (Judgments of Understanding) to track monitoring of memory, learning, and comprehension processes, Aha! ratings may be better considered as “Judgments of Aha!” or JOAs derived from an inferential process based on a variety of cues. Only some of these may directly reflect underlying problem-solving processes. Using a set of spatial object-move puzzles, this study explored JOAs and how they may vary (or fail to vary) with initial fixation, correct solution, and individual differences in growth mindset. Instead of JOAs being highest for solutions where an initial representation needed to be revised, they were highest for solutions where solvers made a correct first move. This suggests that Aha! experiences are more closely tied to achieving correct solutions than to restructuring processes. Individuals with a stronger growth mindset were more likely to associate these initially correct solutions with Aha! experiences.

## 1. Introduction

Insight problem solving is a type of creative problem solving characterized by Gestalt researchers as restructuring, a sudden shift in problem representation that allows the solver to move from an unproductive approach to a productive one (Duncker, 1945; Katona, 1940; Kohler, 1969; Maier, 1931; Ohlsson, 1992; Wertheimer, 1959/2020). Prior to restructuring, problem solvers experience difficulty as they approach a problem using an inappropriate representation. When an initial representation is incorrect and sets the solver down an unproductive path, this can lead the solver to experience fixation in the initial approach or the feeling of being stuck that is called impasse. Following theorizing grounded in a Gestalt approach, it has been suggested that impasse is what leads the problem solver to explore alternative solution approaches, or engage in restructuring of the initial problem representation, in an impasse–insight sequence. This restructuring is thought to be associated with the Aha! moment, the phenomenological experience that a solution appeared to the problem solver suddenly like a light coming on, often accompanied by a rush of positive feelings (Danek et al., 2014; Duncker, 1945; Gick & Lockhart, 1995; Gruber, 1995; Metcalfe, 1986a).

Aha! moments were conceived by the Gestaltists as instances of sudden comprehension that occur simultaneously with restructuring. These moments were thought to be evoked by insightful problem-solving processes in which the solver’s problem representation “flips” to a new understanding which allows the correct solution to emerge, like the reversal in which sides of a Necker Cube appear nearest to the viewer (Laukkonen & Tangen, 2017; Schooler et al., 1995). However, these may not be the only basis for Aha! experiences. Similar to metacognitive paradigms that collect Feelings of Knowing (FOKs) or Judgments of Learning (JOLs) and Judgments of Understanding (JOUs) to track monitoring of memory, learning, and comprehension processes, Aha! ratings may be better considered as “Judgments of Aha!” or JOAs that are inferred based on a variety of cues. Only some of these may directly reflect underlying problem-solving processes.

Metacognition refers to thinking about thinking, typified by internal acts in which we attempt to monitor, evaluate, control and regulate our own cognition (Flavell, 1979). One main approach used in metacognitive research is asking individuals to make ratings on evaluative scales as they engage in cognitive activities. These evaluations are known by various names, but two of the most common are FOKs, when individuals are asked to predict the likelihood of retrieving answers (like definitions or trivia questions) from long-term memory, and JOLs, when individuals are asked to assess their likelihood of recalling recently learned memoranda like word pairs. When these judgments are solicited as predictions before retrieval is attempted on a test, they can be compared to the actual likelihood of retrieval to determine monitoring accuracy or bias. One key theoretical point is that individuals’ assessments of their own cognition can be flawed or biased because they are made indirectly based on a variety of cues. In other words, the process of making FOKs or JOLs is an inferential process, and individuals can use a variety of different sources of information to judge their likely performance. Monitoring accuracy is largely determined by whether the specific cues that are used are valid indicators of memory performance, with cues based in retrieval attempts serving as more diagnostic predictors of later memory (Koriat, 1997; Nelson & Narens, 1990).

Other judgment paradigms have explored monitoring in more complex domains of cognition. It is more difficult to assess one’s own comprehension than memory. In research on metacomprehension, participants make JOCs or JOUs regarding how well they have learned concepts described in passages of text. As the cognitive performance that is being evaluated becomes more complex or ambiguous, there are more ways for monitoring to go wrong, and monitoring accuracy is generally much lower for comprehension than memory tasks. Metamemory judgments can be quite accurate because individuals can engage in retrieval attempts while making their predictions, but also because highly salient cues like fluency tend to be at least somewhat valid predictors of accessibility in memory, which generally predict retrieval. Metacomprehension judgments are less accurate when they rely on these same salient cues (Wiley et al., 2016).

Because the Aha! experience represents a moment of sudden understanding, JOAs can be seen as similar to making JOUs. At the same time, JOAs are also meant to serve as evaluations of how a solution was reached. Instead of assuming that Aha! serves as a marker for a solution that has been achieved via restructuring, it may be better to consider Aha! ratings as metacognitive judgments based on an inferential process which could rely on a variety of cues, including a sense of fluency that can come from the fast retrieval of a solution, or the positive affect that is associated with finding a solution. By this reasoning, there is not a single cause of Aha! experiences, but rather a variety of signals that may give rise to them (Wiley & Danek, 2024).

The goal of this study was to explore possible alternative bases for JOAs by exploring how they may vary (or fail to vary) with initial fixation, correct solution, and individual differences in growth mindset.

### 1.1. Evidence for the Aha!-Insight Link

#### 1.1.1. Insight Memory Advantage

A key finding suggesting a link between Aha! experiences and restructuring is that problems that are solved with Aha! experiences are also associated with better recall of solutions. Katona (1940) and Wertheimer (1959/2020) proposed that this association is because the Aha! experience signals restructuring, the cognitive process that leads to deeper understanding of a problem, while other work shows that generating correct solutions via restructuring leads to better memory for those solutions (Ash & Wiley, 2008; Buyer & Dominowski, 1989; Dominowski & Buyer, 2000). Similarly, Auble and colleagues argued that riddles are better recalled when Aha! experiences occur (Auble et al., 1979; Auble & Franks, 1978). Research using magic tricks and Mooney images as stimuli has demonstrated an *insight memory advantage* for correct solutions that are accompanied by Aha! experiences over those that are not (Danek & Wiley, 2020; Danek et al., 2013; Kizilirmak et al., 2016).

#### 1.1.2. Aha! Accuracy and Correctness Effects

Another key piece of evidence for the link between Aha! experiences and insightful solution processes is that having an Aha! experience generally co-occurs with having reached a correct solution (Danek & Salvi, 2020; Webb et al., 2016; Wiley & Danek, 2024; Zedelius & Schooler, 2015). This correspondence has been referred to as the *Aha!-Accuracy* or *Aha!-Correctness effect* depending on how self-reports of Aha! experiences are collected. The Aha!-Correctness effect is computed when Aha! ratings are collected on continuous scales, where the magnitude of Aha! ratings is compared for correct vs. incorrect answers. The Aha!-Accuracy effect is computed from binary Aha! ratings, where either the likelihood of a correct solution given an Aha! experience or the likelihood of an Aha! experience given a correct solution can be analyzed. Wiley and Danek (2024) reviewed the recent literature in this area and identified evidence for these effects across a variety of problems. They noted that the types of problems that are most often used, and that most often produce these effects tend to be verbal in nature, including remote associate problems based in Mednick (1962) where solvers are asked to find a fourth word that forms a good pair with three other words (Ellis et al., 2021; Laukkonen et al., 2021; Salvi et al., 2016; Shen et al., 2018; Stuyck et al., 2021; Webb et al., 2018, 2019; Zedelius & Schooler, 2015). Effects have also been found for rebus puzzles (Salvi et al., 2016; Threadgold et al., 2018), anagrams (Salvi et al., 2016; Webb et al., 2018), and riddles (Laukkonen et al., 2021; Ross & Vallée-Tourangeau, 2022). Some effects have also been found with nonverbal problems, including identifying how magic tricks are done (Danek et al., 2014, 2020; Danek & Wiley, 2017, 2020; Hedne et al., 2016), and sometimes with matchstick puzzles (Kizilirmak et al., 2021; Strickland et al., 2022). Both the Aha!-Accuracy and Aha!-Correctness effects have been argued to show a “tight coupling” between insight and the Aha! experience (Danek & Salvi, 2020; Threadgold et al., 2018).

#### 1.1.3. Aha! Ratings and Discontinuity in Solution Processes

A third set of findings comes from studies where researchers attempt to test for an association between Aha! experiences and restructuring processes by tracking progress to solution. This approach is similar to Metcalfe’s Feelings of Warmth (FOW) paradigm (Metcalfe, 1986a, 1986b; Metcalfe & Wiebe, 1987) where individuals are asked to make FOW ratings at regular intervals throughout the solution phase. When asked to rate how close they felt to finding a solution on the insight puzzles, Metcalfe found that individuals were unaware when a solution was imminent, whereas they could correctly predict when they were nearing a solution on routine math problems (Metcalfe & Wiebe, 1987). This provided one of the first pieces of evidence suggesting the insight solution process is discontinuous, with solutions emerging suddenly rather than incrementally.

Instead of monitoring proximity to solution, newer studies have asked individuals to rate which elements of a problem seem most important for solution in order to track changes in the participant’s mental representation of a problem (Ash & Wiley, 2008; Cushen & Wiley, 2012; Durso et al., 1994). When repeated throughout the solution phase, these ratings can reveal when solvers become aware of the correct representation needed to solve the problem, and whether movement toward a correct representation is sudden or incremental. In studies that collected both importance-to-solution ratings and Aha! ratings, Danek and colleagues (Danek & Wiley, 2020; Danek et al., 2020) were able to demonstrate that problems solved via discontinuous solution processes resulted in higher Aha! ratings than problems solved via incremental solution processes. In another study, Bilalić et al. (2019) found a similar connection between Aha! ratings and sudden changes in problem representations using data from eye movements to track the solution process.

### 1.2. Disconnect Between Aha! Ratings and Other Insight Measures

At the same time, there have been failures to find these correspondences. Although there have been many studies that have found Aha!-Accuracy or Aha!-Correctness effects, the robustness of the effect varies across paradigms. And most of these effects come from studies using verbal problem types, especially remote associates tasks (Wiley & Danek, 2024). Critically, some studies show sizeable correctness effects on non-insight problems, including logic problems or analytic problems such as items from Raven’s Advanced Progressive Matrices (Laukkonen et al., 2021; Webb et al., 2018). These Aha! effects are similar or greater in size than effects for some problems that are supposed to require restructuring. It should also be noted that even though many of these studies show that Aha! experiences are more likely to be reported for correctly solved problems, they also show that individuals report having Aha! experiences even for incorrect solutions (Strickland et al., 2022; Wiley & Danek, 2024).

Other studies have shown that individuals report having Aha! experiences when no restructuring is required for a solution (Becker et al., 2021; Cranford & Moss, 2012; Webb et al., 2018), or when people fail to generate a correct solution and are just presented with the answer as a revelation (Kizilirmak et al., 2016; Webb et al., 2021). Similarly, solvers are more likely to report Aha! experiences when irrelevant tangential factors are introduced that cause sudden increases in fluency or affect (Grimmer et al., 2022; Laukkonen et al., 2020; Webb et al., 2019). All of these cases of *false insights* are problematic for assuming that Aha! experiences serve as a marker for restructuring (Danek & Wiley, 2017).

Other discrepancies have been found in work that has attempted to track the problem-solving process to determine if restructuring or representational change took place. When insight problems are solved correctly, they do not invariably lead to Aha! moments (Danek et al., 2016). Cushen and Wiley (2012) also tracked whether solution progress was incremental or discontinuous using repeated importance-to-solution ratings and failed to see a relation with Aha! ratings. Other studies with use trace measures have failed to find evidence for a direct connection between restructuring and the Aha! experience (Fedor et al., 2015; Fleck & Weisberg, 2004).

It is difficult to directly equate subjective Aha! experiences with insightful solution processes if strong Aha! experiences are not exclusively linked to generating correct solutions via restructuring. This disconnect between Aha! ratings and other measures of insightful problem solving raises the possibility that other cues may prompt individuals to perceive and report Aha! experiences.

### 1.3. Alternative Possible Bases for Aha! Experiences

When individuals are asked to define Aha! experiences, they report a number of different dimensions including pleasure, confidence, suddenness, surprise, and relief or frustration (Danek et al., 2014). These provide multiple cues that individuals could use to inform their JOAs.

Positive emotions including pleasure, joy, and delight have long been regarded as a hallmark of the Aha! experience (Duncker, 1945; Gick & Lockhart, 1995; Gruber, 1995; Metcalfe, 1986a). However, solvers may experience positive affect simply as a consequence of a solution being generated. The Aha! feeling in this case could be a reaction to having reached a solution and does not provide an indication of how the solution was reached (Wiley & Danek, 2024). This would explain why Aha! experiences can be reported after solutions that are unlikely to have involved restructuring. The positive affect that is associated with the Aha! experience might also serve to boost motivation or curiosity.

Another trigger for reporting an Aha! experience could be the perception of fluency in reaching a solution (Cranford & Moss, 2012; Danek & Wiley, 2017; Salvi et al., 2016; Threadgold et al., 2018; Topolinski & Reber, 2010; Stuyck et al., 2021), even though disfluency (or initial difficulty) should theoretically be more conducive. This would explain why Aha! experiences are reported when solutions are reached immediately.

### 1.4. Individual Differences in Predispositions to Report Aha! Experiences

Prior survey work by Ovington et al. (2018) suggests that Aha! experiences are not universal, with only 80% of their participants reporting they had a previous Aha! experience. In Danek et al. (2016), participants reported experiencing Aha! only about half of the time that they reached correct solutions to classic insight puzzles. Several lines of research have explored increased rates of creative solutions and Aha! experiences among individuals with schizotypic traits such as overinclusive thinking or personality traits such as openness to experience (DiStefano et al., 2025; Kaufman et al., 2016; King et al., 1996; McCrae, 1987; Su et al., 2026; Webb et al., 2021). These results suggest growth mindset as another individual difference that may be of interest. Growth mindset has generally been associated with openness, as well as with enjoyment of challenges and resilience to failure (Dweck, 2006). It is possible that a growth mindset may predispose individuals to report Aha! moments and could inflate their JOAs.

Mindset theory proposes that there are two potential mindsets, fixed and growth. A fixed mindset is the belief that a person’s intelligence and abilities are solid and unchangeable. Certain people are smart while others are not and this is not something that can be changed through any amount of effort. On the other hand, a growth mindset is a belief that a person’s intelligence and abilities are adaptable and can be changed through a person’s efforts. Dweck (2006) highlights two different responses to moments of failure. Those with a growth mindset respond to failure by embracing the opportunity to persist and improve, while those with a fixed mindset give up faster in addition to interpreting failure as a lack of competence (Blackwell et al., 2007).

In the current study, growth mindset was considered as an individual difference variable that could explain why Aha! experiences are more likely or stronger for some people and weaker for others. On one hand, having a growth mindset could serve as a heuristic cue that simply increases the rate of reporting Aha! experiences regardless of the actual underlying solution processes. However, those with a growth mindset could also be better attuned. Similar to Dweck’s observations that some children were excited by challenging problems (Dweck, 2006), having an Aha! experience when solving insight problems has been associated with increased motivation (Danek et al., 2014; Gruber, 1995; Liljedahl, 2005). Additionally, a growth mindset is proposed to enable individuals to respond favorably to failure, and insight problems are specifically designed to lead to initial failure. If those with a growth mindset are more willing to work through failure, it would follow that they would be more likely to benefit from failure, and move past impasse, to restructure and to come to a correct solution (Kaplan & Simon, 1990; Seifert et al., 1995; VanLehn, 1988). For these reasons, growth mindsets might lead to more correct solutions or stronger Aha! experiences following correct solutions.

### 1.5. Current Study

The goal of this study was to explore the extent to which JOAs are linked to insightful solution processes or other factors by exploring how they may vary (or fail to vary) with correct solutions, starting with an incorrect approach and individual differences in growth mindset. The problems used for this study were the four spatial object-move problems from Cushen and Wiley (2012). These puzzles generally lead individuals to start with an incorrect approach that requires restructuring to solve. Considering the first move that solvers attempt can provide evidence of their initial problem representations (Ormerod et al., 2002; Öllinger et al., 2013, 2014). In this study, individuals were asked to report the first move they considered as they first attempted the problem. Then they were given up to 3 min to try to find a correct solution for each problem. After each solution, they made a JOA and rated whether they had an Aha! moment on a 5-point scale.

The study had several hypotheses that tested the assumption that Aha! experiences serve as a marker for insight and assumptions about growth mindsets:

**H1.**  *JOAs should positively relate to performance resulting in an Aha!-Correctness Effect.*

**H2.**  *JOAs should be higher on problems correctly solved after initial difficulty than on those solved correctly immediately.*

**H3.**  *Growth mindset should positively relate to problem-solving performance.*

**H4.**  *Growth mindset should predict higher JOAs following correct solutions.*

## 2. Materials and Methods

### 2.1. Participants and Design

A total of 377 introductory psychology students (*M* = 18.80, *SD* = 1.46) at a large urban university in the United States participated in this study and were given course credit for their participation. Ethics approval for the study was provided by the Institutional Review Board in the Office for the Protection of Research Subjects at the University of Illinois at Chicago (2001-0489).

The majority of the sample was female (68%). The sample identified as 27% Asian, 11% Black, 38% Hispanic/Latinx, 8% Middle Eastern, and 11% White.

This was a repeated-measures design in which all participants completed the same set of 4 problems. Out of 1508 observations (377 individuals × 4 problems), 223 were excluded because individuals reported seeing the problems before, and 120 had missing data representing failures to enter a solution before the deadline, which also meant they had no JOA responses. Although the solution timer was set to 180 s in the data collection program, 89 observations had response times between 180 and 240 s, presumably due to server lags. These cases were retained in analyses. The final sample included 1164 observations for analysis.

### 2.2. Materials

#### 2.2.1. Insight Problems

**Figure 1:**
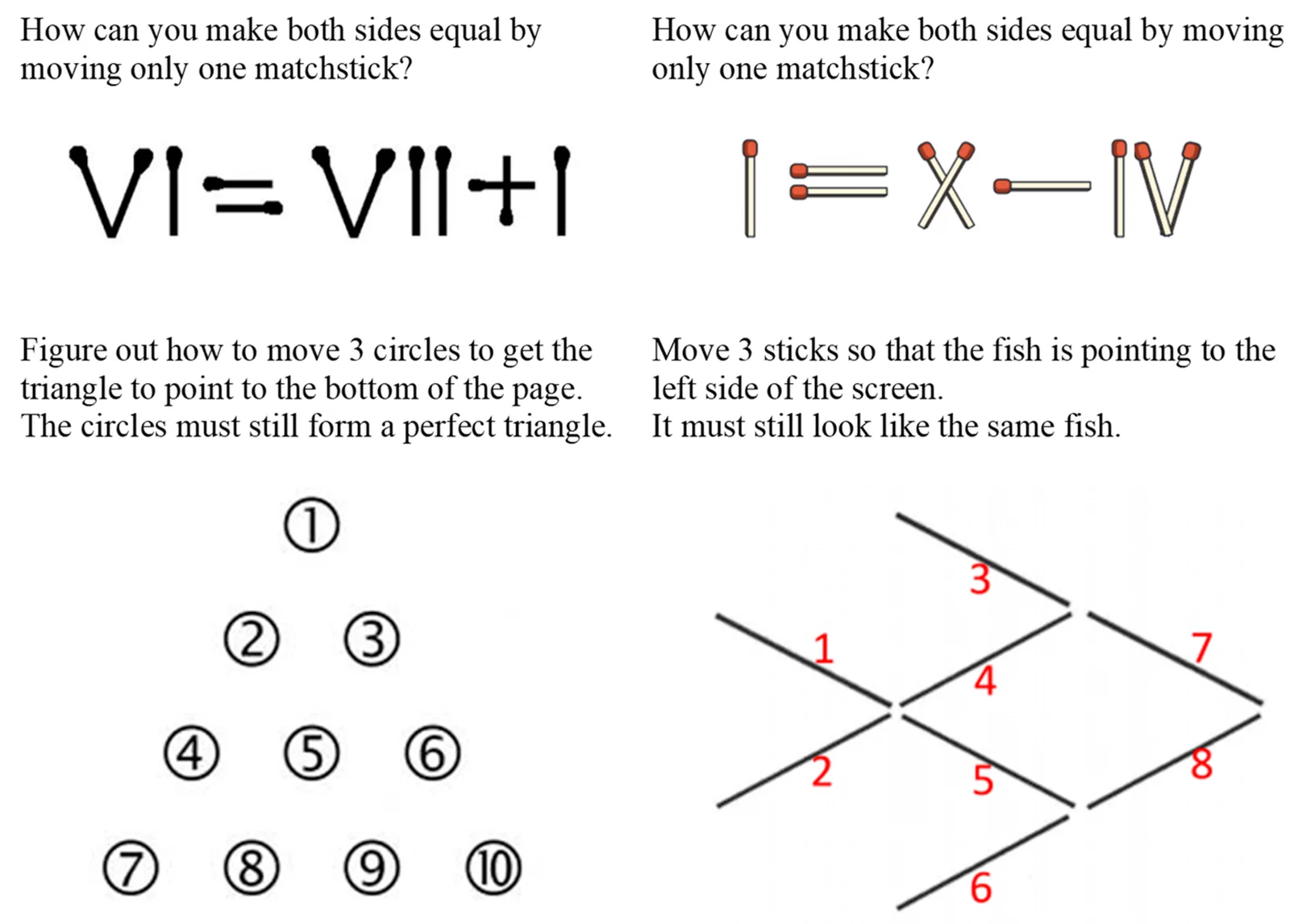
Matchstick arithmetic, triangle of coins, and matchstick fish problems.

The 4 problems involved moving one or more objects to achieve a new goal (rotating the shape or making a correct mathematical statement). These problems are presented in Figure 1, and the full instructions given to solvers are included on OSF (see Data Availability Statement). (The numerals did not appear on the fish problem; they are included so solutions can be discussed below).

Two problems were matchstick arithmetic problems that are solved by moving a single matchstick to turn an incorrect mathematical statement into a correct one (Knoblich et al., 1999, 2001). The first matchstick problem (Matchstick 1) required decomposition of a loose chunk by moving a single vertical matchstick from VII to VI. A common incorrect answer is trying to move the single I to the left-hand side and leaving the extra +. The second matchstick problem (Matchstick 2) required decomposition of a tight chunk by sliding one of the two matchsticks that form either the X or V into a V or X. A common incorrect approach is to try to move the single stick in the IV on the right to form a V on the left.

The other two problems, triangle of coins (or circles) and matchstick fish (Cushen & Wiley, 2012), involve moving three items, circles or matchsticks, to flip the direction of the shape from top to bottom for the triangle problem and right to left for the fish problem. For the triangle, a common incorrect approach is to try to move the top three circles (1, 2, 3) to the bottom. The correct answer requires moving 1, 7, and 10, so that the top row has 4 circles and the new bottom row has 1. For the fish, a common incorrect approach is to try to move the “nose” or the “tail” to the opposite side, or to try flipping the direction of the top and bottom fins. The correct answer is either shifting all three top sticks down (1, 3, 7 so that the 1 completes the “head” on the bottom left, 3 becomes the bottom fin, and 7 becomes the bottom tail) or to shift all three bottom sticks up (2, 6, 8 so that the head is on the top left).

#### 2.2.2. Judgments of Aha! (JOAs)

After entering a solution for each problem, participants were asked to respond to the following JOA prompt: “If you found a solution to this problem, how much did it feel like an Aha! moment?”. The response options were a Likert scale from 1–5, from Not at All to Very Much. No additional information about Aha! experiences. No definition of an Aha! experience was provided to the participants during the study.

#### 2.2.3. Dweck Mindset Inventory

The Dweck Mindset Inventory (DMI, Dweck, 1999) was used to assess participants’ self-perceptions of their mindset. The DMI included 16 questions answered on a 1–6 scale from Strongly Agree to Strongly Disagree. Three scores were computed following the procedure outlined in Barbouta et al. (2020): an overall DMI score, a DMI intelligence subscore, and a DMI ability subscore. Cronbach’s alpha was .72. Overall, participants did not show a difference across the three ways to score the DMI measure. The continuous ability subscore of DMI was used for all mindset analyses since the ability to solve problems was deemed most relevant for this problem solving task.

### 2.3. Procedure

This study was conducted using a Qualtrics survey. The main part of the study entailed answering the 4 problems within a 3 min time limit. Participants were specifically told:

“You will now be asked to respond to some problems. Please read the instructions for each problem carefully. Each problem will be presented for 3 min only and then the survey will automatically continue. After the survey has moved on you will be given the opportunity to input your answer to the problem. Click the arrow when you are ready to begin with the first problem.”

As soon as they finished reading each problem, they were asked to enter the first move they thought of into an open-ended textbox: “Describe the first move you try in the box below.” Then they had up to 3 min to solve the problem. If they found a solution before time ran out, they were asked to click the arrow when they knew the answer. On the next screen, they entered their solution by selecting which of the objects needed to be moved. To enable multiple-choice responses, each object in the figure was numbered so that individuals could select the options from a list. If a solution was entered, then the JOA was solicited before presenting the next problem. No feedback was given for any of the problems.

In a final survey, participants were asked whether they had seen any of the problems before, as well as demographic questions about their age, gender, and race. The DMI was collected in a separate prescreening survey.

### 2.4. Data Analysis Plan

Key variables of interest were participants’ open-ended responses in which they described the first move they attempted on each problem (scored for accuracy), their final solutions of which objects needed to be moved from the multiple-choice list (scored for accuracy), and their JOAs. For simplicity, because the data were normally distributed and the results were the same, JOA results were analyzed with linear mixed effects (LME) models rather than ordinal/cumulative link mixed models (Norman, 2010). Because each participant contributed 4 data points to the data set, the main analyses were performed using mixed models using GAMLj3 (Gallucci, 2025) in JAMOVI (The Jamovi Project, 2025, Version 2.6.44). When there was a significant interaction with another fixed effect and problem, then problem was included as a fixed effect. Otherwise, random intercepts were included for both participants and problems. The graphs were created with ggplot2 (Wickham, 2016). Confidence intervals for the computed effect sizes were estimated using Soper (2026). Interrater reliability was computed using the seolmatrix module (Seol, 2025).

### 2.5. Open Science Availability

All materials, the data set, and analyses are available on OSF (See Data Availability Statement).

## 3. Results

Average solution rates and JOAs are presented for each problem in Table 1.

### 3.1. Testing for the Aha!-Correctness Effect

To test for the Aha!-Correctness effect, an LME analysis tested whether correct solutions were associated with higher JOAs. The model included correctness of solution, problem number, and their interaction as fixed effects with random intercepts for participants, *R*^2^ = .415. Overall, JOAs were higher for correct solutions, *F*(1, 1091) = 112.17, *p* < .001, although there was a significant effect of problem *F*(3, 894) = 21.02, *p* < .001, and a significant interaction between correctness and problem, *F*(3, 1021) = 9.84, *p* < .001. As shown in Table 1, the average total Aha! ratings were lowest on the Fish problem, and highest on the Triangle problem and Matchstick Problem 1. In addition, the interaction was due to smaller differences between average JOAs for correct and incorrect solutions on the Matchstick problems than the Triangle and Fish problems, although simple effects tests showed that all problems showed a significant Aha!-Correctness Effect. To determine the effect size for the correctness fixed effect, the full model was compared to a reduced model that excluded this predictor, *R*^2^_partial_ = .09, 95% CI [.06, .12], Cohen’s *f*^2^ = 0.099.

### 3.2. Testing for Associations Between Aha! Ratings and Restructuring

Theoretically, if Aha! experiences reflect a change in representation or restructuring during problem solving attempts that allow the correct solution to suddenly emerge, then individuals who do not already know the correct solution approach at the start of problem solving, but who eventually solve the problem, should experience the strongest Aha! experiences. To test this hypothesis, the open-ended responses that individuals provided to describe their first moves were scored based on whether or not the first move was consistent with a correct problem representation. A first move was scored as correct when it listed the same correct objects (and no incorrect objects) as required for the final solution responses. Kappa for two independent coders was .94. The proportions (and frequencies) of correct final solutions for both first-move categories are shown in Table 1. Interestingly, although these problems have been used to study the insight process, the likelihood of reaching a correct solution after starting on an incorrect path was relatively uncommon.

**Figure 2:**
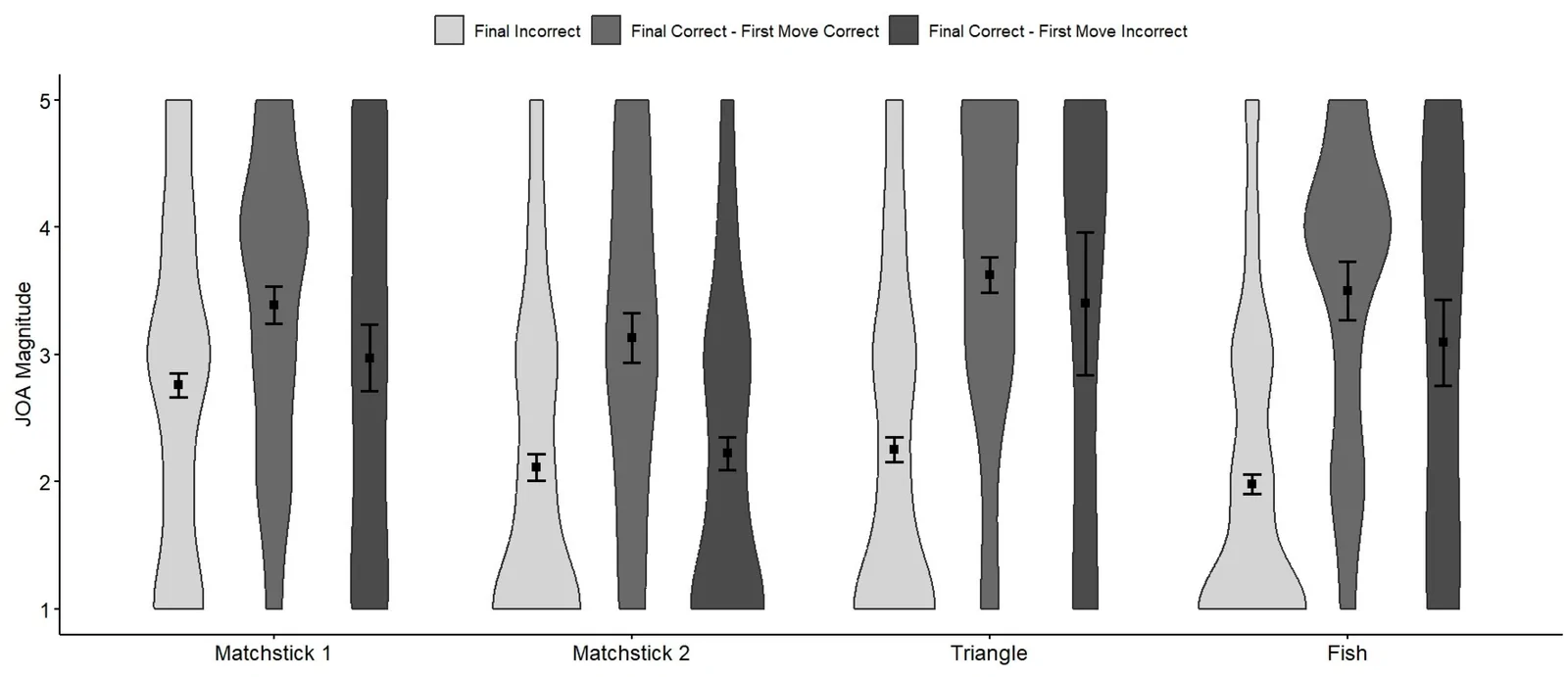
Aha-Accuracy effects for all 4 problems, split by incorrect final solutions, correct final solutions reached after a correct first move, and correct final solutions reached after an incorrect first move. Error bars represent standard error.

An LME analysis tested if correct final solutions reached after starting with an incorrect representation were associated with higher JOAs than correct final solutions where solvers made a correct first move or reached incorrect final solutions. This model included an index representing these three levels of correctness of solution, problem number, and their interaction as fixed effects with random intercepts for participants. As shown in Figure 2, a significant effect was seen for the correctness index, *F*(2, 1079) = 71.48, *p* < .001. Aha! ratings were lowest for incorrect final solutions. But, instead of JOAs being highest for problems where an initial representation needed to be revised, they were highest for correct final solutions where solvers made a correct first move. This suggests that Aha! experiences were more closely tied to achieving correct solutions than to restructuring processes.

There was again a main effect for problem, *F*(2, 945) = 10.08, *p* < .001, and also a significant interaction, *F*(2, 1007) = 4.54, *p* < .001. Again, the two matchstick problems were more similar to each other in their patterns of means, with both showing only Aha!-Correctness effect for correct final solutions reached after a correct first move, and no effect for correct final solutions reached after a change in approach compared to incorrect final solutions. And again, the Triangle and Fish problems were more similar to each other, with both showing an Aha!-Correctness effect for both types of correct final solutions over incorrect final solutions. Importantly, correct final solutions reached after an incorrect first move never resulted in the highest Aha! ratings. To determine the effect size for the correctness fixed effect, the full model was compared to a reduced model that excluded this predictor, *R*^2^_partial_ = .11, 95% CI [.08, .14], Cohen’s *f*^2^ = 0.126.

### 3.3. Effects of Growth Mindset on Problem-Solving Performance

The first analysis on growth mindset tested if DMI scores predicted problem-solving performance. The logistic mixed-effects regression model included growth mindset (average DMI ability score) as a fixed effect, and random intercepts for participant and problem. For the DMI score, higher values indicate a growth mindset while lower values indicate a fixed mindset. Overall, growth mindset did not predict the likelihood of reaching a correct solution (*B* = −0.04, *SE* = 0.07, *Z* = −0.48, *p* = .632).

A second analysis explored persistence on problems by looking at solution times for correct and incorrect responses. The linear mixed-effects model included correctness of solution, DMI score, and their interaction as fixed effects, with random intercepts for participants and problems, and showed that solution time increased with growth mindset, *F*(1, 355) = 25.07, *p* < .001, regardless of whether they reached correct or incorrect solutions. Neither the effect of solution correctness nor the interaction was significant. To determine the effect size for growth mindset, the full model was compared to a reduced model that excluded this predictor, *R*^2^_partial_ = .04, 95% CI [.02, .06], Cohen’s *f*^2^ = 0.043.

### 3.4. Effects of Growth Mindset on Aha! Ratings

**Figure 3:**
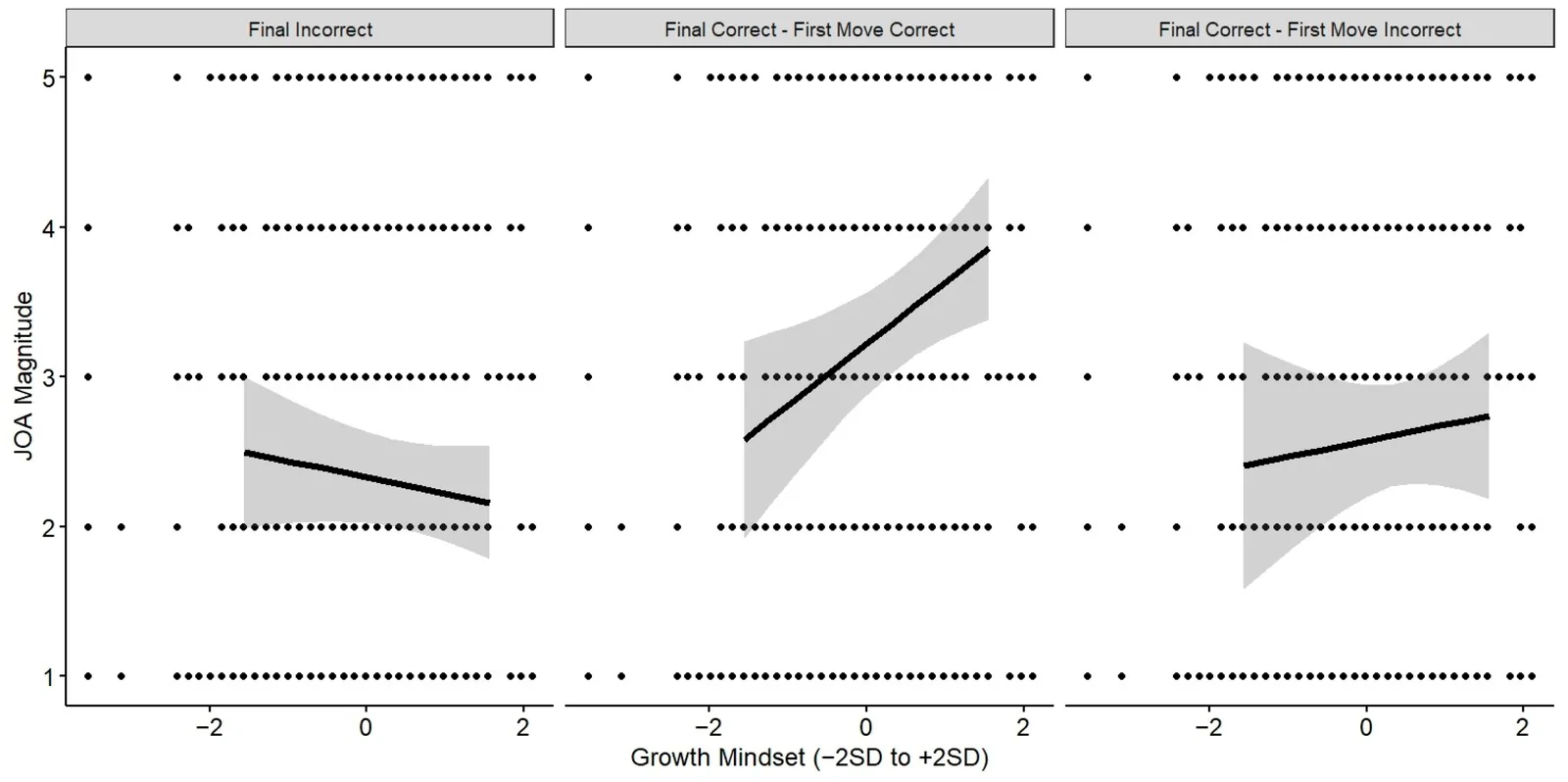
Effects of growth mindset on JOAs for incorrect final solutions, correct final solutions reached after a correct first move, and correct final solutions reached after an incorrect first move. Shaded areas represent 95% confidence bands.

Finally, a linear mixed-effects model including the three-level correctness of solution index, DMI score, and their interaction as fixed effects along with random intercepts for participants and problems showed no significant effect of DMI score on JOA magnitude. However, it did reveal a significant interaction between DMI score and the correctness index, *F*(2, 1071) = 5.13, *p* = .006. As shown in Figure 3, individuals with higher DMI scores (meaning stronger growth mindsets) were the ones most likely to report higher JOAs for correct final solutions that followed a correct first move over incorrect final solutions. They did not, however, show increases in JOAs for the correct final solutions that were reached following incorrect first moves over incorrect final solutions. Again, this suggests that Aha! experiences are more closely tied to achieving correct solutions than to restructuring processes, but in addition, this analysis shows that individuals with growth mindsets are more likely to report Aha! experiences in association with these correct solutions. To determine the effect size for the interaction, the full model was compared to a reduced model that excluded this predictor, *R*^2^_partial_ = .01, 95% CI [−.00, .02], Cohen’s *f*^2^ = 0.007.

## 4. Discussion

Overall, Aha! experiences were positively associated with correct solutions, consistent with prior work showing Aha!-Accuracy and Aha!-Correctness effects. This finding extends work on Aha!-Correctness effects to a new set of problems. Previous work has primarily shown these effects using verbal problems, while work with nonverbal problems has only shown robust effects for magic trick stimuli. The results for matchstick problems have been mixed, with one study showing effects (Kizilirmak et al., 2021) but not another (Strickland et al., 2022). Two of these spatial problems (Fish and Triangle) showed robust effects, but consistent with prior work, the effects on the matchstick puzzles were weaker. As suggested in Wiley and Danek (2024), this variability, especially between verbal and nonverbal problem types, may be due in part to different kinds of restructuring that may be needed for these problems.

The increase in studies exploring Aha! moments over the past two decades has been largely prompted by early work with compound remote associates derived from Mednick’s task (Bowden & Jung-Beeman, 2003). From those verbal puzzles, work has spread to a wide variety of contexts. For example, there are now studies that have documented Aha! moments in resolving polysemy (Smith et al., 2025), disambiguating garden path sentences (Dygert & Jarosz, 2026), and engaging in divergent thinking during category generation tasks (Smith et al., 2026). Aha! moments have also been found to accompany conceptual change (Chesebrough et al., 2023). Not only might these different contexts involve different types of restructuring, but they could also give rise to different kinds of Aha! experiences.

A key contribution from this study was showing that Aha! effects were not the same for all solutions. Theoretically, if Aha! experiences reflect a change in representation or restructuring during problem-solving attempts that allow the correct solution to suddenly emerge, then individuals who do not already know the correct solution approach at the start of problem solving, but who eventually solve the problem, should report the strongest JOAs. However, the first-move analyses revealed that the Aha!-Correctness effect was being driven by correct solutions that followed an already correct first move, and *not* by those that followed an incorrect first move. If incorrect-first-move solutions are ones that are most likely to require representational change, this result suggests that higher JOAs are more likely based on reactions of pleasure, joy, or satisfaction at having found a solution, or feelings of fluency from having found a solution immediately, rather than having reached a solution via restructuring (Wiley & Danek, 2024). Consistent with considering Aha! ratings as metacognitive judgments that reflect an inferential process, individuals appear to use these salient affective and fluency-based cues to inform their JOAs. However, this interpretation assumes that the first-move analysis provides a good measure of when restructuring should be required. It cannot rule out that restructuring might have already occurred even before the first move was reported.

The results also documented that correct solutions that followed an incorrect first move were relatively infrequent. This finding is consistent with other work that has found that the impasse–insight solution sequence may be quite rare and often does not account for the majority of solutions, even on problems that are typically used to study insight problem solving (Ash et al., 2012; Fleck & Weisberg, 2004, 2013). This means that any work attempting to study insight needs to be sure to collect measures such as first moves, importance-to-solution ratings, or other trace measures that can allow researchers to assess whether solvers are achieving solutions via restructuring. Solution rates alone are not adequate to ensure that one is studying insightful problem solving, as many solutions to these puzzles appear to be reached without the signature initial difficulties that require insight.

A final contribution of the study was investigating whether individual differences in growth mindset might determine who experiences Aha! moments during problem solving. In this study, there was no overall effect of growth mindset on solution rates. Nor was there an effect on the overall magnitude of Aha! experiences, suggesting that a strong growth mindset does not prompt individuals to perceive or report Aha! moments indiscriminately. However, growth mindset did affect persistence in the amount of time that individuals spent on the problems, and it amplified Aha! ratings specifically for problems that were solved correctly from the outset. Although the impact of a growth mindset on academic outcomes has been brought into question (Burgoyne et al., 2020; Macnamara & Burgoyne, 2023; Sisk et al., 2018), these results align with claims that a growth mindset may help individuals to be curious or motivated, and to respond positively to challenging problem solving tasks.

## 5. Conclusions

The current study provides evidence of the *Aha-Correctness effect* with spatial object-move problems. At the same time, it replicates prior results by finding that two matchstick arithmetic problems showed weaker effects. More critically, this study used a “first move” measure to try to discriminate correct solutions that were reached with and without initial difficulty. Instead of solutions that were found after an incorrect initial attempt, it was solutions that were found immediately that led to the highest JOAs. Further, individuals with a stronger growth mindset were more likely to associate these initially correct solutions with Aha! experiences.

## Figures and Tables

**Table 1:** Solution rates (and frequencies) and average JOAs by problem and solution type.

Problem	Correct Solutions	Average JOA for All Solutions	Average JOA for Correct Solutions	Average JOA for Incorrect Solutions	Correct First Move Solutions	Wrong First Move Solutions
Matchstick 1	33%	2.92	3.24	2.76	21% (66)	12% (37)
Matchstick 2	48%	2.31	2.52	2.11	16% (45)	33% (95)
Triangle	34%	2.71	3.60	2.25	29% (78)	05% (12)
Fish	17%	2.21	3.33	1.98	10% (29)	07% (20)

## Data Availability

All materials, the data set, and analyses are available on OSF at https://osf.io/ctuvh/overview?view_only=432c3510b9b6438ca50027d361814d22 (accessed on 20 July 2026).

## Abbreviations

The following abbreviations are used in this manuscript:

DMI	Dweck Mindset Inventory
FOK	Feeling of Knowing
FOW	Feeling of Warmth
JOA	Judgment of Aha!
JOL	Judgment of Learning
JOU	Judgment of Understanding

## References

[B1-jintelligence-14-00198] Ash I. K., Jee B. D., Wiley J. (2012). Investigating insight as sudden learning. Journal of Problem Solving.

[B2-jintelligence-14-00198] Ash I. K., Wiley J. (2008). Hindsight bias in insight and mathematical problem solving: Evidence of different reconstruction mechanisms for metacognitive versus situational judgments. Memory & Cognition.

[B3-jintelligence-14-00198] Auble P. M., Franks J. J. (1978). The effects of effort toward comprehension on recall. Memory & Cognition.

[B4-jintelligence-14-00198] Auble P. M., Franks J. J., Soraci S. A. (1979). Effort toward comprehension: Elaboration or “aha”?. Memory & Cognition.

[B5-jintelligence-14-00198] Barbouta A., Barbouta C., Kotrotsiou S. (2020). Growth mindset and grit: How do university students’ mindsets and grit affect their academic achievement?. International Journal of Caring Sciences.

[B6-jintelligence-14-00198] Becker M., Kühn S., Sommer T. (2021). Verbal insight revisited—Dissociable neurocognitive processes underlying solutions accompanied by an Aha! experience with and without prior restructuring. Journal of Cognitive Psychology.

[B7-jintelligence-14-00198] Bilalić M., Graf M., Vaci N., Danek A. H. (2019). The temporal dynamics of insight problem solving—Restructuring might not always be sudden. Thinking & Reasoning.

[B8-jintelligence-14-00198] Blackwell L. S., Trzesniewski K. H., Dweck C. S. (2007). Implicit theories of intelligence predict achievement across an adolescent transition: A longitudinal study and an intervention. Child Development.

[B9-jintelligence-14-00198] Bowden E. M., Jung-Beeman M. (2003). Aha! Insight experience correlates with solution activation in the right hemisphere. Psychonomic Bulletin & Review.

[B10-jintelligence-14-00198] Burgoyne A. P., Hambrick D. Z., Macnamara B. N. (2020). How firm are the foundations of mind-set theory? The claims appear stronger than the evidence. Psychological Science.

[B11-jintelligence-14-00198] Buyer L. S., Dominowski R. L. (1989). Retention of solutions: It is better to give than to receive. The American Journal of Psychology.

[B12-jintelligence-14-00198] Chesebrough C., Chrysikou E. G., Holyoak K. J., Zhang F., Kounios J. (2023). Conceptual change induced by analogical reasoning sparks Aha moments. Creativity Research Journal.

[B13-jintelligence-14-00198] Cranford E. A., Moss J. (2012). Is insight always the same? A protocol analysis of insight in compound remote associate problems. The Journal of Problem Solving.

[B14-jintelligence-14-00198] Cushen P. J., Wiley J. (2012). Cues to solution, restructuring patterns, and reports of insight in creative problem solving. Consciousness & Cognition.

[B15-jintelligence-14-00198] Danek A. H., Fraps T., Von Müller A., Grothe B., Öllinger M. (2013). Aha! experiences leave a mark: Facilitated recall of insight solutions. Psychological Research.

[B16-jintelligence-14-00198] Danek A. H., Fraps T., Von Müller A., Grothe B., Öllinger M. (2014). It’s a kind of magic—What self-reports can reveal about the phenomenology of insight problem solving. Frontiers in Psychology.

[B17-jintelligence-14-00198] Danek A. H., Salvi C. (2020). Moment of truth: Why Aha! experiences are correct. Journal of Creative Behavior.

[B18-jintelligence-14-00198] Danek A. H., Wiley J. (2017). What about false insights? Deconstructing the Aha! experience along its multiple dimensions for correct and incorrect solutions separately. Frontiers in Psychology.

[B19-jintelligence-14-00198] Danek A. H., Wiley J. (2020). What causes the insight memory advantage?. Cognition.

[B20-jintelligence-14-00198] Danek A. H., Wiley J., Öllinger M. (2016). Solving classical insight problems without Aha! experience: 9 dot, 8 coin, and matchstick arithmetic problems. Journal of Problem Solving.

[B21-jintelligence-14-00198] Danek A. H., Williams J., Wiley J. (2020). Closing the gap: Connecting sudden representational change to the subjective Aha! experience in insightful problem solving. Psychological Research.

[B22-jintelligence-14-00198] DiStefano P. V., Patterson J. D., Beaty R. E. (2025). Evaluating overinclusive thinking: Development and validation of the Categorical Overinclusive Thinking Task (COverTT). Thinking Skills and Creativity.

[B23-jintelligence-14-00198] Dominowski R. L., Buyer L. S. (2000). Retention of problem solutions: The re-solution effect. The American Journal of Psychology.

[B24-jintelligence-14-00198] Duncker K. (1945). On problem-solving (L. S. Lees, Trans.). Psychological Monographs.

[B25-jintelligence-14-00198] Durso F. T., Rea C. B., Dayton T. (1994). Graph-theoretic confirmation of restructuring during insight. Psychological Science.

[B26-jintelligence-14-00198] Dweck C. S. (1999). Self-theories: Their role in motivation, personality, and development.

[B27-jintelligence-14-00198] Dweck C. S. (2006). Mindset: The new psychology of success.

[B28-jintelligence-14-00198] Dygert S. K. C., Jarosz A. F. (2026). Navigating the garden path: Evidence for restructuring as a shared process. Journal of Intelligence.

[B29-jintelligence-14-00198] Ellis D. M., Robison M. K., Brewer G. A. (2021). The cognitive underpinnings of multiply-constrained problem solving. Journal of Intelligence.

[B30-jintelligence-14-00198] Fedor A., Szathmáry E., Öllinger M. (2015). Problem solving stages in the five square problem. Frontiers in Psychology.

[B31-jintelligence-14-00198] Flavell J. H. (1979). Metacognition and cognitive monitoring: A new area of cognitive–developmental inquiry. American Psychologist.

[B32-jintelligence-14-00198] Fleck J. I., Weisberg R. W. (2004). The use of verbal protocols as data: An analysis of insight in the candle problem. Memory & Cognition.

[B33-jintelligence-14-00198] Fleck J. I., Weisberg R. W. (2013). Insight versus analysis: Evidence for diverse methods in problem solving. Journal of Cognitive Psychology.

[B34-jintelligence-14-00198] Gallucci M. (2025). GAMLj3: GAMLj Suite for linear models *[jamovi module]*.

[B35-jintelligence-14-00198] Gick M. L., Lockhart R. S., Sternberg R. J., Davidson J. E. (1995). Cognitive and affective components of insight. The nature of insight.

[B36-jintelligence-14-00198] Grimmer H., Laukkonen R., Tangen J., von Hippel W. (2022). Eliciting false insights with semantic priming. Psychonomic Bulletin & Review.

[B37-jintelligence-14-00198] Gruber H. E., Sternberg R. J., Davidson J. E. (1995). Insight and affect in the history of science. The nature of insight.

[B38-jintelligence-14-00198] Hedne M. R., Norman E., Metcalfe J. (2016). Intuitive feelings of warmth and confidence in insight and noninsight problem solving of magic tricks. Frontiers in Psychology.

[B39-jintelligence-14-00198] Kaplan C. A., Simon H. A. (1990). In search of insight. Cognitive Psychology.

[B40-jintelligence-14-00198] Katona G. (1940). Organizing and memorizing: Studies in the psychology of learning and teaching.

[B41-jintelligence-14-00198] Kaufman S. B., Quilty L. C., Grazioplene R. G., Hirsh J. B., Gray J. R., Peterson J. B., DeYoung C. G. (2016). Openness to experience and intellect differentially predict creative achievement in the arts and sciences. Journal of Personality.

[B42-jintelligence-14-00198] King L. A., Walker L. M. K., Broyles S. J. (1996). Creativity and the five-factor model. Journal of Research in Personality.

[B43-jintelligence-14-00198] Kizilirmak J. M., Gallisch N., Schott B. H., Folta-Schoofs K. (2021). Insight is not always the same: Differences between true, false, and induced insights in the matchstick arithmetic task. Journal of Cognitive Psychology.

[B44-jintelligence-14-00198] Kizilirmak J. M., Galvao Gomes da Silva J., Imamoglu F., Richardson-Klavehn A. (2016). Generation and the subjective feeling of “Aha!” are independently related to learning from insight. Psychological Research.

[B45-jintelligence-14-00198] Knoblich G., Ohlsson S., Haider H., Rhenius D. (1999). Constraint relaxation and chunk decomposition in insight problem solving. Journal of Experimental Psychology: Learning, Memory, and Cognition.

[B46-jintelligence-14-00198] Knoblich G., Ohlsson S., Raney G. E. (2001). An eye movement study of insight problem solving. Memory & Cognition.

[B47-jintelligence-14-00198] Kohler W. (1969). The task of Gestalt psychology.

[B48-jintelligence-14-00198] Koriat A. (1997). Monitoring one’s own knowledge during study: A cue-utilization approach to judgments of learning. Journal of Experimental Psychology: General.

[B49-jintelligence-14-00198] Laukkonen R. E., Ingledew D. J., Grimmer H. J., Schooler J. W., Tangen J. M. (2021). Getting a grip on insight: Real-time and embodied Aha experiences predict correct solutions. Cognition and Emotion.

[B50-jintelligence-14-00198] Laukkonen R. E., Kaveladze B. T., Tangen J. M., Schooler J. W. (2020). The dark side of Eureka: Artificially induced Aha moments make facts feel true. Cognition.

[B51-jintelligence-14-00198] Laukkonen R. E., Tangen J. M. (2017). Can observing a Necker cube make you more insightful?. Consciousness and Cognition.

[B52-jintelligence-14-00198] Liljedahl P. G. (2005). Mathematical discovery and affect: The effect of Aha! experiences on undergraduate mathematics students. International Journal of Mathematical Education in Science and Technology.

[B53-jintelligence-14-00198] Macnamara B. N., Burgoyne A. P. (2023). Do growth mindset interventions impact students’ academic achievement? A systematic review and meta-analysis with recommendations for best practices. Psychological Bulletin.

[B54-jintelligence-14-00198] Maier N. R. F. (1931). Reasoning in humans. II. The solution of a problem and its appearance in consciousness. Journal of Comparative Psychology.

[B55-jintelligence-14-00198] McCrae R. R. (1987). Creativity, divergent thinking, and openness to experience. Journal of Personality and Social Psychology.

[B56-jintelligence-14-00198] Mednick S. A. (1962). The associative basis of the creative process. Psychological Review.

[B57-jintelligence-14-00198] Metcalfe J. (1986a). Feeling of knowing in memory and problem solving. Journal of Experimental Psychology: Learning, Memory, and Cognition.

[B58-jintelligence-14-00198] Metcalfe J. (1986b). Premonitions of insight predict impending error. Journal of Experimental Psychology: Learning, Memory, & Cognition.

[B59-jintelligence-14-00198] Metcalfe J., Wiebe D. (1987). Intuition in insight and noninsight problem solving. Memory & Cognition.

[B60-jintelligence-14-00198] Nelson T. O., Narens L. (1990). Metamemory: A theoretical framework and new findings. Psychology of Learning and Motivation.

[B61-jintelligence-14-00198] Norman G. (2010). Likert scales, levels of measurement and the “laws” of statistics. Advances in Health Sciences Education.

[B62-jintelligence-14-00198] Ohlsson S., Keane M. T., Gilhooly K. J. (1992). Information-processing explanations of insight and related phenomena. Advances in the psychology of thinking.

[B63-jintelligence-14-00198] Ormerod T. C., MacGregor J. N., Chronicle E. P. (2002). Dynamics and constraints in insight problem solving. Journal of Experimental Psychology: Learning, Memory, and Cognition.

[B64-jintelligence-14-00198] Ovington L. A., Saliba A. J., Moran C. C., Goldring J., MacDonald J. B. (2018). Do people really have insights in the shower? The when, where and who of the Aha! moment. The Journal of Creative Behavior.

[B65-jintelligence-14-00198] Öllinger M., Jones G., Faber A. H., Knoblich G. (2013). Cognitive mechanisms of insight: The role of heuristics and representational change in solving the eight-coin problem. Journal of Experimental Psychology: Learning, Memory, and Cognition.

[B66-jintelligence-14-00198] Öllinger M., Jones G., Knoblich G. (2014). Insight and search in Katona’s five-square problem. Experimental Psychology.

[B67-jintelligence-14-00198] Ross W., Vallée-Tourangeau F. (2022). Insight with stumpers: Normative solution data for 25 stumpers and a fresh perspective on the accuracy effect. Thinking Skills and Creativity.

[B68-jintelligence-14-00198] Salvi C., Bricolo E., Kounios J., Bowden E., Bowman M. (2016). Insight solutions are correct more often than analytic solutions. Thinking & Reasoning.

[B69-jintelligence-14-00198] Schooler J. W., Fallshore M., Fiore S. M., Sternberg R. J., Davidson J. E. (1995). Epilogue: Putting insight into perspective. The nature of insight.

[B70-jintelligence-14-00198] Seifert C. M., Meyer D. E., Davidson N., Patalano A. L., Yaniv I., Sternberg R. J., Davidson J. E. (1995). Demystification of cognitive insight: Opportunistic assimilation and the prepared-mind perspective. The nature of insight.

[B71-jintelligence-14-00198] Seol H. (2025). Seolmatrix: A module for calculating correlations and agreement in jamovi *(Version 4.0.1) [jamovi module]*.

[B72-jintelligence-14-00198] Shen W., Tong Y., Yuan Y. (2018). Feeling the insight: Uncovering somatic markers of the “aha” experience. Applied Psychophysiology and Biofeedback.

[B73-jintelligence-14-00198] Sisk V. F., Burgoyne A. P., Sun J., Butler J. L., Macnamara B. N. (2018). To what extent and under which circumstances are growth mind-sets important to academic achievement? Two meta-analyses. Psychological Science.

[B74-jintelligence-14-00198] Smith S. M., Chandolia V. J., Kidd M. A., Paladino M. S. (2026). Generative insight: Aha! Moments in category generation and divergent thinking. Journal of Experimental Psychology: General.

[B75-jintelligence-14-00198] Smith S. M., Kidd M. A., Chandolia V. J., Hermoso J. M. (2025). Verbal insight problems and Aha! moments. Creativity Research Journal.

[B76-jintelligence-14-00198] Soper D. S. (2026). R-square confidence interval calculator *[Software]*.

[B77-jintelligence-14-00198] Strickland T., Wiley J., Ohlsson S. (2022). Hints and the aha-accuracy effect in insight problem solving. Proceedings of the annual meeting of the cognitive science society.

[B78-jintelligence-14-00198] Stuyck H., Aben B., Cleeremans A., Van den Bussche V. (2021). The Aha! moment: Is insight a different form of problem solving?. Consciousness and Cognition.

[B79-jintelligence-14-00198] Su L. C., Wei J. H., Chuang H. H. (2026). Fostering divergent thinking through a growth creative mindset: The mediating roles of multicultural attitudes and openness to experience. Thinking Skills and Creativity.

[B80-jintelligence-14-00198] The Jamovi Project (2025). Jamovi *(Version 2.6.44) [Computer Software]*.

[B81-jintelligence-14-00198] Threadgold E., Marsh J. E., Ball L. J. (2018). Normative data for 84 UK English rebus puzzles. Frontiers in Psychology.

[B82-jintelligence-14-00198] Topolinski S., Reber R. (2010). Gaining insight into the “Aha” experience. Current Directions in Psychological Science.

[B83-jintelligence-14-00198] VanLehn K., Mandl H., Lesgold A. M. (1988). Toward a theory of impasse-driven learning. Learning issues for intelligent tutoring systems.

[B84-jintelligence-14-00198] Webb M. E., Cropper S. J., Little D. R. (2019). “Aha!” is stronger when preceded by a “huh?”: Presentation of a solution affects ratings of aha experience conditional on accuracy. Thinking & Reasoning.

[B85-jintelligence-14-00198] Webb M. E., Little D. R., Cropper S. J. (2016). Insight is not in the problem: Investigating insight in problem solving across tasktypes. Frontiers in Psychology.

[B86-jintelligence-14-00198] Webb M. E., Little D. R., Cropper S. J. (2018). Once more with feeling: Normative data for the Aha experience in insight and noninsight problems. Behavior Research Methods.

[B87-jintelligence-14-00198] Webb M. E., Little D. R., Cropper S. J. (2021). Unusual uses and experiences are good for feeling insightful, but not for problem solving: Contributions of schizotypy, divergent thinking, and fluid reasoning, to insight moments. Journal of Cognitive Psychology.

[B88-jintelligence-14-00198] Wertheimer M. (2020). Productive thinking.

[B89-jintelligence-14-00198] Wickham H. (2016). ggplot2: Elegant graphics for data analysis.

[B90-jintelligence-14-00198] Wiley J., Danek A. H. (2024). Restructuring processes and Aha! experiences in insight problem solving. Nature Reviews Psychology.

[B91-jintelligence-14-00198] Wiley J., Thiede K. W., Griffin T. D., Mokhtari K. (2016). Improving metacomprehension with the situation-model approach. Improving reading comprehension through metacognitive reading instruction for first and second language readers.

[B92-jintelligence-14-00198] Zedelius C. M., Schooler J. W. (2015). Mind wandering “Ahas” versus mindful reasoning: Alternative routes to creative solutions. Frontiers in Psychology.

